# Thyroid Storm-Induced Acute Liver Dysfunction and Disseminated Intravascular Coagulation

**DOI:** 10.7759/cureus.16504

**Published:** 2021-07-20

**Authors:** Yungmin Kim, Dawa Gurung, Vikram Sumbly, David M Reich, Tayyaba Bashir

**Affiliations:** 1 Internal Medicine, Icahn School of Medicine at Mount Sinai, Queens Hospital Center, New York, USA; 2 Medicine/Endocrinology, Icahn School of Medicine at Mount Sinai, Queens Hospital Center, New York, USA; 3 Hematology and Medical Oncology, Icahn School of Medicine at Mount Sinai, Queens Hospital Center, New York, USA

**Keywords:** thyrotoxicosis, graves disease, disseminated intravascular coagulation, thrombocytopenia, hepatitis

## Abstract

Thyroid storm-induced disseminated intravascular coagulation (DIC) is a very rare complication of untreated/undertreated Graves' disease. It is considered to be a medical emergency as it can rapidly lead to hemodynamic instability in patients due to multi-organ failure. Although the exact pathogenesis of this hematological phenomenon remains poorly understood, it is believed to be triggered by the uncontrolled release of pro-inflammatory cytokines, which in turn prematurely activates the coagulation cascade. In this report, we present the case of a 48-year-old female who presented with symptoms of abdominal pain, dyspnea, and unintentional weight loss for the past several weeks. Her vital signs, overall clinical picture, and laboratory tests confirmed thyroid storm complicated by DIC and acute liver injury. The patient made a significant recovery after the initiation of methimazole therapy.

## Introduction

Graves’ disease remains the most common cause of hyperthyroidism worldwide. It is an endocrinological disease characterized by the auto-immune overstimulation of the thyroid gland [1]. Although Graves’ disease is more common in young adult females, it can occur in both sexes at any age [1]. Patients with Graves’ disease often experience mood fluctuations, weight loss, increased hunger, tremors, palpitations, diaphoresis, diarrhea, and heat intolerance [1]. If left untreated or severely undertreated, Graves’ disease can progress to thyroid storm, an uncommon form of decompensated Graves’ disease. Thyroid storm is considered to be a medical emergency as it is associated with an increased risk of morbidity and mortality. This life-threatening complication can lead to hyperpyrexia, myocardial dysfunction, pulmonary edema, and metabolic encephalopathy. In very rare instances, thyroid storms can also induce disseminated intravascular coagulation (DIC), a serious hematological syndrome that is characterized by the premature activation of the coagulation cascade [2]. Thus, patients with thyroid storms complicated by DIC often bear a poor prognosis as they can rapidly go into multi-organ failure. Diagnosis is often clinical but needs to be corroborated by several laboratory tests and imaging studies. The prompt initiation of anti-thyroid medications has been shown to significantly decrease the risk of thyroid storm complications.

## Case presentation

A 48-year-old female with a past medical history of hypertension and “thyroid dysfunction” (patient unaware of the details) came to the emergency department with complaints of ongoing dyspnea and right upper abdominal pain for the past several days. She had also experienced an unintentional 15 lbs weight loss over the course of four months. On admission, the patient’s blood pressure (BP) was 156/95 mmHg, pulse was 104 beats per minute (bpm), the temperature was 100.3 °F (37.9 °C), respiratory rate was 18 breaths per minute, and oxygen saturation was 96% on room air. The physical examination was only significant for a diffuse petechial rash on both upper extremities. The initial laboratory results were significant for anemia, severe thrombocytopenia, and transaminitis (Table 1).

**Table 1:** Initial labs from the emergency room WBC: white blood cells; Hgb: hemoglobin; MCV: mean corpuscular volume; ALT: alanine aminotransferase; AST: aspartate aminotransferase; BUN: blood urea nitrogen

Variables (reference range)	Results	Variables (reference range)	Results
WBC (4.80-10.80 x 10³/mcL)	6.21	Serum albumin (3.5-5.2 g/dL)	2.8
Hgb (12.0-16.0 g/dL)	9.2	Total protein (6.6-8.7 g/dL)	6.7
MCV (80.0-99.0 fL)	64.2	Total bilirubin (0.0-1.2 mg/dL)	0.9
Platelets (150-450 x 10³/mcL)	29	Alkaline phosphatase (35-104 U/L)	166
Schistocyte	Slight	ALT (0-33 U/L)	151
Respiratory viral panel	Not detected	AST (5-32 U/L)	446
Lipase (13-60 U/L)	59	BUN (6-23 mg/dL)	11
Sodium (136-145 mmol/L)	137	Creatinine (0.5-1.2 mg/dL)	0.42
Potassium (3.5-5.1 mmol/L)	3.7	Chloride (98-108 mmol/L)	106
CO_2_ (22-29 mmol/L)	21	Anion gap (8-16 mEq/L)	10

Hematology was consulted, and they recommended obtaining a peripheral smear, which later revealed the presence of multiple schistocytes. Post-admission lab work was significant for iron deficiency anemia, worsening transaminitis, and hyperthyroidism (Tables 2, 3, 4). Furthermore, fibrinogen and haptoglobin levels were found to be decreased (Table 2). Direct antiglobulin test (DAT) and reticulocyte index were within normal limits (Table 2).

The patient then developed a fever of 104.1 °F (40.0 °C) and was immediately started on broad-spectrum antibiotics. A CT angiography (CTA) of the lungs showed no signs of pulmonary embolus or pneumonia but did show a prominent thyroid isthmus. A CT and ultrasound of the abdomen showed nonspecific gall bladder thickening but were negative for common bile duct thickening. There was no growth in blood and urine cultures.

The patient underwent pre-emptive plasmapheresis as her PLASMIC score was 5/7. The bone marrow biopsy result was nonspecific and showed mild hypercellular marrow (60-70%), with trilineage hematopoiesis, with proportionate increases in myeloid and erythroid lineages, both of which appeared mildly left-shifted. Fibrinogen levels improved after plasmapheresis (Table 5). Plasmapheresis was discontinued once the a disintegrin and metalloproteinase with a thrombospondin type 1 motif, member 13 (ADAMTS13) assay was within normal limits.

Endocrinology recommended starting the patient on a stress dose of glucocorticoids, cholestyramine, propranolol, and supersaturated potassium. Once the patient’s liver function test improved, she was started on methimazole, which decreased her free T3 to 4.0 ng/dl and free T4 to 2.1 ng/dl (Table 4).

The patient was finally diagnosed with thyroid storm secondary to untreated Graves’ disease complicated by DIC and acute liver dysfunction. The patient was discharged after 11 days of hospitalization as her clinical condition and laboratory results normalized.

**Table 2:** Labs after the admission TSH: thyroid-stimulating hormone; TSI: thyroid-stimulating immunoglobulin; PT: prothrombin time; aPTT: activated partial thromboplastin time; LDH: lactate dehydrogenase; TIBC: total iron-binding capacity; IgM: immunoglobulin M; HBV: hepatitis B virus; INR: international normalized ratio; ALT: alanine aminotransferase; AST: aspartate aminotransferase

Variables (reference range)	Results	Variables (reference range)	Results
Free T4 (0.9-1.8 ng/dL)	6.5	HBV surface antigen	Nonreactive
Free T3 (1.8-4.6 ng/dL)	6.11	HBV core antibody	Nonreactive
TSH (0.27-4.2 μIU/mL)	<0.01	HEV antibody	Nonreactive
TSI (0-0.55 IU/L)	15.20	HAV antibody	Nonreactive
Fibrinogen (200-400 mg/dL)	78	HCV antibody	Nonreactive
Fibrin split products (<5 μg/mL)	≥20	HIV antigen/antibody screen	Nonreactive
PT (10.0-13.0 sec)	24.8	INR (ratio)	2.1
aPTT (27.0-36.0 sec)	36.3	Serum albumin (3.5-5.2 g/dL)	2.7
LDH (135-214 U/L)	>900	Total protein (6.6-8.7 g/dL)	6.4
Haptoglobin (34-200 mg/dL)	<20	Total bilirubin (0.0-1.2 mg/dL)	2.6
Iron saturation (14-50%)	12	Alkaline phosphatase (35-104 U/L)	153
TIBC (220-430 μg/dL)	266	ALT (0-33 U/L)	878
Ferritin (15-150 ng/mL)	2,189	AST (5-32 U/L)	2,676
Reticulocytes (0.5-1.5%)	2.07	Acetaminophen (10-30 μg/mL)	<5
Mycoplasma IgM	Negative		

**Table 3:** Follow-up labs ALT: alanine aminotransferase; AST: aspartate aminotransferase; ADAMTS13: a disintegrin and metalloproteinase with a thrombospondin type 1 motif, member 13; LDH: lactate dehydrogenase; Hgb: hemoglobin

Variables (reference range)	Results	Variables (reference range)	Results
Serum albumin (3.5-5.2 g/dL)	2.5	ADAMTS13, %	81.3
Total protein (6.6-8.7 g/dL)	6.1	LDH (135-214 U/L)	1,634
Total bilirubin (0.0-1.2 mg/dL)	2.9	Haptoglobin (34-200 mg/dL)	<20
Alkaline phosphatase (35-104 U/L)	144	Pre-plasmapheresis fibrinogen (200-400 mg/dL)	59
ALT (0-33 U/L)	607		
AST (5-32 U/L)	1,367	Hgb (12.0-16.0 g/dL)	7.8
Ceruloplasmin (16-45 mg/dL)	26	Platelets (150-450 x 10³/mcL)	10

**Table 4:** Liver function test during the hospital course ALT: alanine aminotransferase; AST: aspartate aminotransferase

Liver function test									
Admission days	Day 2	Day 3	Day 4	Day 6	Day 7	Day 8	Day 9	Day 10	Day 11
Albumin (3.5-5.2 g/dL)	2.5	3.4	4.2	3.5	3.4	3.4	3.5	3.8	3.1
Total protein (6.6-8.7 g/dL)	6.1	6.2	7.5	5.8	5.6	5.6	6	6	5.1
Total bilirubin (0.0-1.2 mg/dL)	2.9	2.6	3	2.1	1.1	0.8	0.8	0.7	0.7
Direct bilirubin (0.0-0.3 mg/dL)	2.2	1.5	1.1	1	0.6	0.4	0.3	0.3	0.3
Alkaline phosphatase (35-104 U/L)	144	104	108	93	90	84	101	99	82
ALT (0-33 U/L)	607	214	118	67	48	31	50	45	32
AST (5-32 U/L)	1,367	364	187	64	43	32	45	35	25

**Table 5:** Fibrinogen level after the plasmapheresis

Variable (reference range)	Result
Day 2 post-plasmapheresis fibrinogen (200-400 mg/dL)	196

## Discussion

Based on our patient’s vague history of “thyroid dysfunction” and lack of medical follow-up, she went into thyrotoxicosis crisis due to untreated Graves’ disease. Although a diagnosis of thyroid storm is based on clinical features, the Burch-Wartofsky Point Scale (BWPS) can be used to determine the likelihood of thyroid storm in adults with underlying thyrotoxicosis [3]. Upon admission, our patient had a score of 25 on the BWPS scoring system, which indicated an impending thyroid storm. Her score increased to 45 once she had a fever of 104.1 °F, which was strongly suggestive of a thyroid storm. An abnormal thyroid function test and CT scan further confirmed our suspicions; the thyroid function test revealed ongoing hyperthyroidism, whereas the CT scan showed a prominent thyroid isthmus. Prompt intervention is required when there is high suspicion for thyroid storm because mortality rates vary between 10-30% [2,4].

The pathogenesis of thyroid storm-induced DIC with acute liver injury remains poorly understood as there are very few case reports linking these three entities [5-9]. Our patient could have developed DIC due to severe inflammation, coagulation factor imbalances, and tissue damage [10].

Thyroid storms have been associated with an excessive release of pro-inflammatory cytokines via systemic inflammatory response syndrome (SIRS) [11]. Studies have shown that thyroid hormones are essential for blood coagulation. Indeed, thyroxine is known to cause platelet activation and induce the production of pro-inflammatory cytokines such as interleukin-1 (IL-1). IL-1 creates a pro-thrombotic environment by increasing the production of tissue factor (TF), large von Willebrand factor (VWF) multimers, IL-6, IL-8, and other adhesion molecules [6].

Our patient’s worsening transaminitis indicated hepatocellular injury. The mechanism of liver dysfunction in thyrotoxicosis is not clear, but one animal study suggests that severe hyperthyroidism can induce apoptosis through various mitochondrial pathways by increasing the activation of caspase 3 and 9 [12]. When DIC is associated with liver injury, it is often due to decreased levels of fibrinogen, plasminogen, and vitamin K-dependent coagulation factors (II, VII, IX, and X). Surprisingly, most patients do not bleed until they experience end-stage liver disease [10].

## Conclusions

Although the association between thyroid storm and DIC has yet to be fully elucidated, it is evident that thyroid hormones have a vital role in the coagulation pathway. It is unclear if our patient’s DIC was a direct consequence of her thyroid storm or was a by-product of her acute hepatic injury. Nonetheless, it is important to keep thyroid storm-induced DIC in mind whenever a patient with uncontrolled Graves’ disease presents with severe hematological abnormalities.
